# Recurrent Small Bowel Obstruction Related to Cryptogenic Multifocal Ulcerating Stenosing Enteropathy

**DOI:** 10.14309/crj.0000000000001390

**Published:** 2024-07-11

**Authors:** Jack Mlabasati, Kara Raphael, Marc Greenwald, Deepika Savant, Keith Sultan

**Affiliations:** 1 Division of Gastroenterology & Hepatology, Zucker School of Medicine at Hofstra/Northwell, Northshore University Hospital, Manhasset, NY

**Keywords:** small bowel ulcerations, cryptogenic multifocal ulcerating stenosing enteropathy, cryptogenic multifocal stenosing ulceration, non-inflammatory small bowel disease

## Abstract

Cryptogenic multifocal ulcerating stenosing enteropathy is a rare idiopathic small bowel enteropathy characterized by multiple small intestinal strictures and superficial ulcerations, often with clubbing. We present a case of a 25-year-old man who originally initially presented with small bowel obstruction believed to be secondary to suspected Crohn's disease who was ultimately diagnosed with cryptogenic multifocal ulcerating stenosing enteropathy.

## INTRODUCTION

Small bowel ulcerations and obstructions are frequently encountered during routine gastroenterology clinical practice. Although Crohn's disease (CD) may be the first cause that comes to mind, the differential diagnosis is relatively broad and includes infectious etiologies such as mycobacterium, syphilis, typhoid, and histoplasmosis, as well as medication induced from nonsteroidal anti-inflammatory drug (NSAID) or potassium supplementation, neoplasm, and other inflammatory etiologies such as Bechet's disease, celiac disease, vasculitis, and radiation enteritis.^1^ We present a case of a 25-year-old man who presented with small bowel obstruction believed to be secondary to CD who was ultimately diagnosed with cryptogenic multifocal ulcerating stenosing enteropathy (CMUSE).

## CASE REPORT

**Figure 1.**
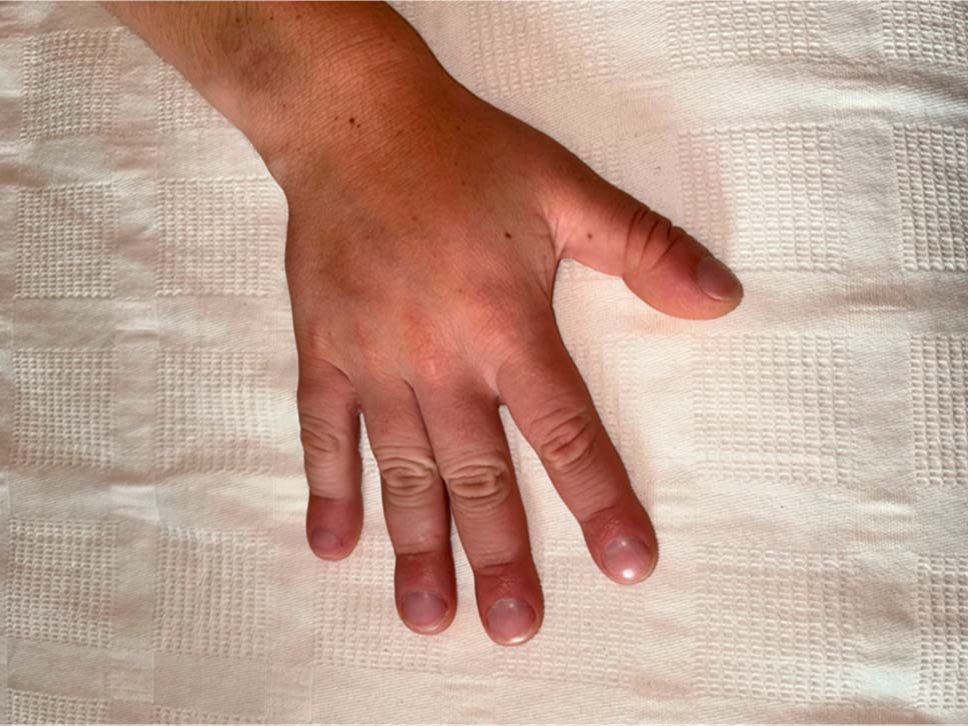
Prominent clubbing of the patients' fingers.

A 25-year-old man presented to our clinic with a history of chronic diarrhea, seronegative spondyloarthropathy, iron deficiency anemia, protein-losing enteropathy, recurrent small bowel obstructions, and elevated fecal calprotectin level to 278 μg/g. History and subsequent physical examination revealed no oral aphthous or genital ulcers and ascites, although note was made of prominent clubbing of the fingers bilaterally (Figure 1). Blood testing revealed a negative Quantiferon gold result and negative celiac serologies. Given the suspicion for CD, the patient underwent a magnetic resonance enterography that showed some mild prominence of jejunal loops without focal abnormality or areas of mucosal enhancement. A subsequent esophagogastroduodenoscopy with push enteroscopy and colonoscopy was unremarkable. With CD remaining the leading differential diagnosis, the patient was subsequently trialed on ustekinumab.

**Figure 2.**
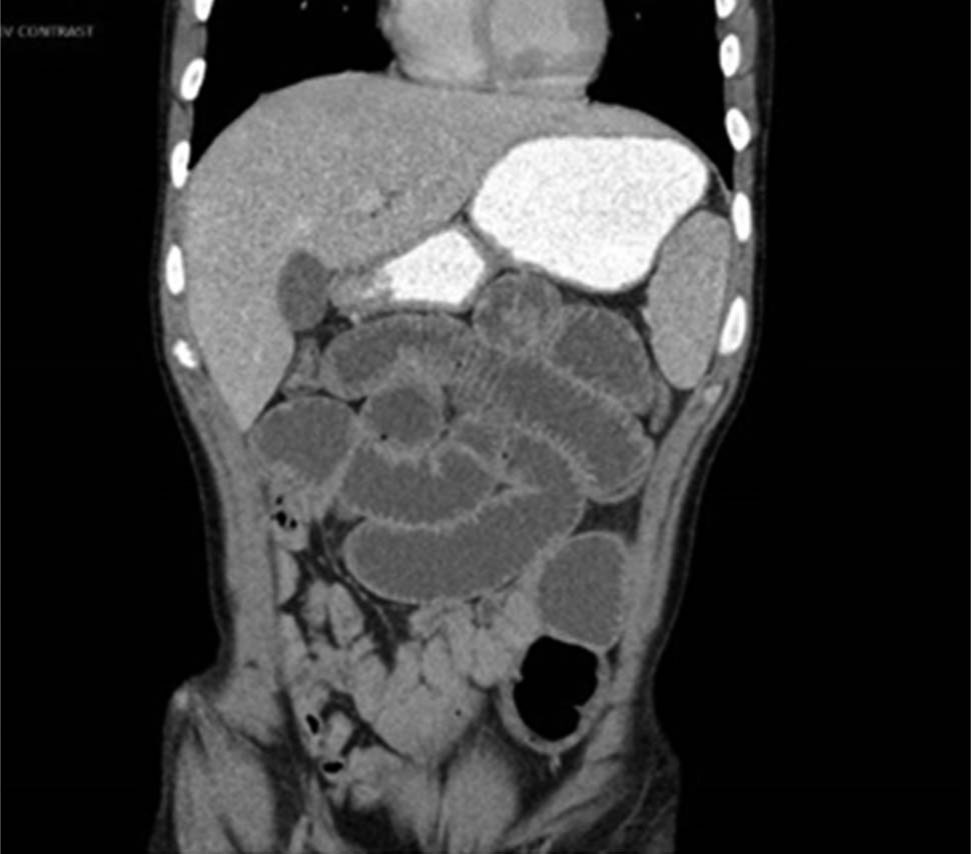
Coronal computed tomography scan showing an acute noninflammatory small bowel obstruction.

**Figure 3.**
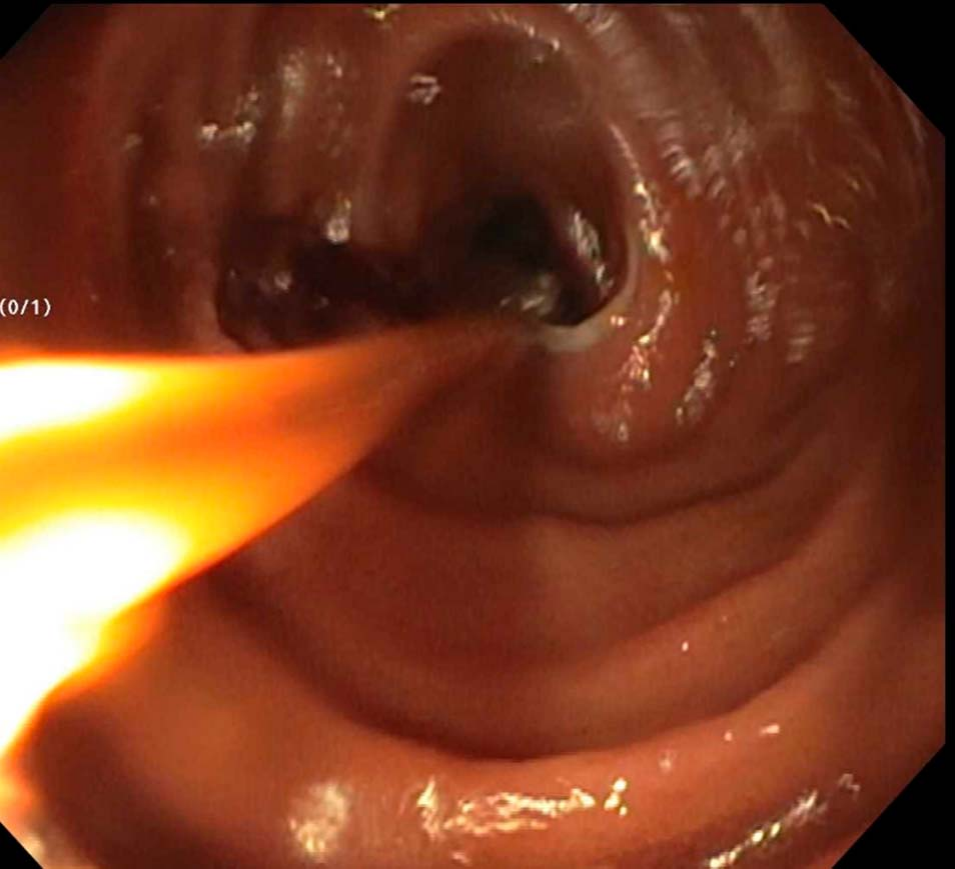
Small bowel balloon enteroscopy showing a benign-appearing non–inflammatory-appearing stenoses in the distal small bowel.

**Figure 4.**
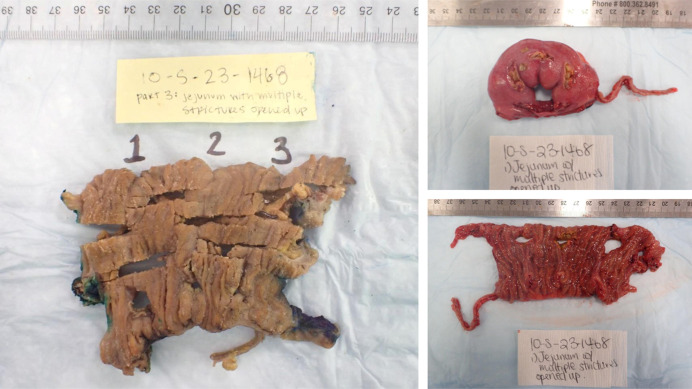
Histopathologic examination showing a segment of small bowel shows multiple well-delineated mucosal ulcers, ranging from 4 mm to less than a millimeter. These are formed of fibrinopurulent exudate with underlying granulation tissue. The intervening mucosa shows no changes of previous or chronic mucosal injury.

Despite some clinical improvement after 15 months of therapy with ustekinumab, mainly with decreased stool frequency and urgency, the patient continued to have elevated inflammatory markers and subsequently presented to the hospital with an acute noninflammatory small bowel obstruction, which improved with nasogastric tube decompression (Figure 2). To further workup his underlying symptoms, the patient underwent a video capsule which revealed numerous stenoses. A small bowel balloon enteroscopy also showed multiple non–inflammatory-appearing stenoses in the distal small bowel (Figure 3). Given recurrent obstructive symptoms, a laparoscopy was performed revealing multiple jejunal strictures without creeping fat or thickening of the mesentery. After a longitudinal enterotomy, a pediatric colonoscope was threaded proximally and distally revealing 8 strictures separated by at least 10 cm, allowing for stricturoplasties. Another segment with 5 strictures, all within several centimeters, was resected. Surgical pathology showed multiple superficial mucosal ulcers, fibrinopurulent exudate with intervening mucosa showing no changes of previous or chronic mucosal injury or evidence of granulomas, transmural inflammation, pyloric metaplasia, dysplasia, or viral cytopathic effect and not suggestive of small bowel Crohn's disease (Figure 4). The patient denied any underlying NSAID use, supporting a diagnosis of CMUSE. Although not clinically pathognomonic, the patient was noted to have clubbing of his fingers, suggesting a chronic enteropathy associated with *SCLCO2A1* gene, which is a subtype of CMUSE (Figure 1). The patient overall felt clinically well after his small bowel resection with no recurrent episodes of small bowel obstructions since his small bowel resection.

Given the clinical rarity of this clinical condition and lack of clear consensus guidelines on how to optimally manage this condition, the patient will undergo serial monitoring with monitoring of serum and stool inflammatory markers including C-reactive protein and fecal calprotectin levels in addition to serial radiographic and endoscopic monitoring with alternating computed tomography enteropathy and video capsule endoscopy.

## DISCUSSION

The differential diagnosis of patients presenting with small bowel ulcerations include infections, CD, and a drug-induced enteropathy such as NSAIDs. Rarely, patients may develop an uncommon form of a small bowel enteropathy such as CMUSE or cryptogenic multifocal ulcerous stenosing enteritis (CNSU).^1^ CMSU/CNSU is a rare idiopathic small bowel enteropathy first described in Europe and Asia in the 1950s characterized by multiple small intestinal strictures and superficial ulcerations often mimicking NSAID-induced enteropathy or CD. Patients can often present with unexplained small bowel strictures, with multiple, circular, or eccentric oblique superficial shallow ulcerations extending to the submucosa that can present clinically as chronic diarrhea, recurrent small bowel obstruction, and occult blood loss with no signs of systemic inflammation.^2^ Symptoms tend to be relapsing and remitting with medical therapy including corticosteroids, which may help reverse underlying strictures and ulcerations. Given the overall chronic disease course, patients may often develop steroid dependence. There have been case reports describing the use of infliximab to help with the above.^3^

Although the etiology of CNSU was believed to be idiopathic in nature, there may be a genetic etiology, leading to the development of the above.^4^ Notably, loss of function mutations in *PLA2G4A* and *SLCO2A1*, genes that are responsible in encoding a prostaglandin transporter, have been found in patients with CMUSE/CNSU.^5^ Specifically, the *SLCO2A1* gene is responsible for producing the *SLCO2A1* protein, which is expressed on the cellular membrane of vascular endothelial cells in the small intestinal mucosa. This protein is involved in mediating the uptake and clearance of prostaglandins with loss of function mutations leading to the development of ulcers and strictures.

Interestingly, some patients with primary hypertrophic osteoarthropathy have also been found to have defects in the *SLCO2A1* gene.^6,7^ These patients can often develop with clinical features including digital clubbing, periostosis, and pachydermia. As noted in Figure 4, our patient was noted to have prominent digital clubbing, leading to the suspicion of this rare subtype of CNSU believed to be related to a loss of function mutation of a *SLCO2A1* gene.

In conclusion, we demonstrate a case of structuring small bowel disease related to CMUSE/CNSU mimicking a pre-existing diagnosis of Crohn's disease. Although objectively rare, physicians should keep CMUSE/CNSU of the differential when encountering patients with multiple small bowel ulcers. Last, further studies are warranted to elucidate the optimal management strategies and preventive measures for patients with CMUSE/CNSU.

## DISCLOSURES

Author contributions: J. Mlabasati and K. Sultan were responsible for conception, design, and drafting of the article. K. Raphael, M. Greenwald, and D. Savant provided analysis and critical revision of the article. All authors were involved in the final review and approval of the article. K. Sultan is the article guarantor.

Financial disclosure: K. Raphael currently serves as a consultant to Olympus. The other authors have no conflict of interest to declare.

Informed consent was obtained for this case report.
